# Precision Medicine in the Diagnosis and Management of Orthopedic Biofilm Infections

**DOI:** 10.3389/fmed.2020.580671

**Published:** 2020-11-10

**Authors:** Rossella Baldan, Parham Sendi

**Affiliations:** ^1^Institute for Infectious Diseases, University of Bern, Bern, Switzerland; ^2^Centre for Musculoskeletal Infections, University Hospital Basel, Basel, Switzerland; ^3^Division of Infectious Diseases and Hospital Epidemiology, Departments of Medicine and Clinical Research, University Hospital Basel, Basel, Switzerland; ^4^Department of Orthopaedic and Trauma Surgery, University Hospital Basel, Basel, Switzerland

**Keywords:** biofilms, osteomyelitis, implant-related infection, minimal biofilm eradication concentration, next-generation sequencing, metagenomic sequencing

## Abstract

Orthopedic biofilm infections are difficult to treat and require a multidisciplinary approach to diagnostics and management. Recent advances in the field include methods to disrupt biofilm, sequencing tools, and antibiotic susceptibility tests for bacteria residing in biofilm. The observation of interclonal differences in biofilm properties of the causative microorganisms, together with considerations of comorbidities and polypharmacy in a growing aging population, calls for a personalized approach to treat these infections. In this article, we highlight aspects of precision medicine that may open new perspectives in the diagnosis and management of orthopedic biofilm infections.

## Introduction

Biofilms generally form on a nonliving surface. In bone and joint infections, microorganisms adhere either to dead bone (sequesters) or to implants. Hence, orthopedic biofilm infections include chronic osteomyelitis and implant-associated infections. They represent a serious threat for the patient and a substantial burden on the global health care industry. More than a million knee and hip arthroplasties are performed every year in the United States (1). Projections for arthroplasties in the United States show—in comparison to 2000 to 2014—an increase by 75, 129, and 284% (hips) and by 110, 182, and 401% (knees) in 2025, 2030, and 2040, respectively (2). Periprosthetic joint infections (PJIs) occur in 0.3–1.7% of patients after total replacement of the hip, in 0.5–2% after total replacement of the knee, and in 2–9% after total replacement of the ankle (3). The incidence of surgical site infection following open reduction and internal fixation of a fracture of the extremities is 1–5% (4). In addition, the implant can be infected via the hematogenous route as long as it remains in the body (5, 6). These arguments underscore the importance of the endeavor to constantly advance research on bone and joint infections.

Biofilm development plays a pivotal role in implant-associated infections and allows microorganisms to survive in an environment protected by antimicrobial agents and the host immune system (7). Successful management of orthopedic biofilm infections eradicates the infection while preserving a pain-free functional musculoskeletal apparatus (with or without implant). This is best achieved by combining an appropriate surgical procedure with antimicrobial treatment and by considering the characteristics of each individual patient.

An optimal and individualized approach considers several factors, including stability of the implant, causative pathogen and type of infection, patient's concomitant comorbidities, and surgical procedure limitations. The concept of “precision medicine” is the new frontier of the modern health care industry and it combines multiple fields of expertise (e.g., genetics, genomics, big data analytics, and population health) (8). It recognizes individual variability in genes, environment, and lifestyle for each person. Precision medicine aims to replace the classic “one-size-fits-all” therapeutic strategy and to achieve “the right drug, with the right dose at the right time to the right patient” (9). By identifying patients most likely to benefit from a specific treatment, clinical outcomes can be improved and side effects and costs reduced. To achieve this, precision medicine must rely on accurate diagnostic tools to effectively maximize benefits and reduce risks to patients.

In orthopedic biofilm infections, precision medicine applies to biofilm properties of the causative microorganisms. Identifying factors associated with so-called low or high biofilm production can influence treatment strategies. The ability of bacteria to adhere to nonliving surfaces and the antibiotic susceptibility patterns of biofilm—together with host and surgical factors—may aid decision making regarding implant removal. In this article, we examine diagnostic advances in the identification of causative microorganisms and antimicrobial susceptibility testing of biofilm bacteria, putting into perspective how these methods can help individualize the management of bone and joint infections.

## Advances in the Diagnosis of Orthopedic Biofilm Infections

Culture of bone or peri-implant tissue samples is the gold standard for identifying the organism causing the infection. Low-frequency ultrasound (i.e., sonication) is a useful tool for the clinical microbiologist in the diagnosis of biofilm-associated infections. Culture of sonication fluid increases the sensitivity and microbiological yield by disrupting the bacterial biofilm and is less affected by prior antimicrobial treatment than are prosthetic tissue samples (10, 11).

### Molecular Diagnostic Tests

Molecular diagnostic tests directly applicable to clinical samples have rapidly developed in recent years, improving the sensitivity of PJI diagnosis. They can be applied to both tissue and sonication samples. Table 1 provides an overview of different molecular techniques evaluated for the diagnosis of PJI, and displays their corresponding sensitivity and specificity. Reported sensitivities of PCR assays applied to DNA extracted from sonication fluid range from 70 to 96% (12–15). Broad-range PCR assays can identify organisms present in the panel, but will miss atypical, rare, and nontargeted pathogens (34). Assays targeting the universal 16S rRNA gene followed by sequencing allowed researchers to partially overcome this limit. Although, the different regions that can be chosen as the target and the limited resolution among closely related species still represent a barrier. This obstacle can be tackled by next-generation sequencing (NGS)-based assays. The term NGS, also known as massive parallel sequencing, refers to non-Sanger-based high throughput DNA sequencing technologies, that allow millions of small or large (depending on the use of short or long read-based technologies) DNA fragments to be sequenced and deciphered simultaneously and independently (35). NGS can be applied using either a targeted or untargeted approach. Targeted NGS focuses on selected portions of genome, while untargeted NGS adopts an unbiased, hypothesis-free approach to detect any portion of genome. In clinical microbiology, after its introduction, NGS was initially applied to cultured isolates to obtain the complete DNA sequence of their genome at a single time (whole genome sequencing, WGS). More recently, the method was applied directly to clinical samples, allowing the identification of pathogens and prediction of antimicrobial resistance (36–42). When applied directly to clinical samples using an untargeted approach, NGS allows the comprehensive characterization of all nucleic acids (microbial and human) present in the sample. This approach is called untargeted metagenomic NGS (mNGS) (43). This technique can significantly reduce the turnaround time to diagnosis compared with culture methods and can detect pathogens not identified by conventional methods (38–41).

**Table 1:** Overview of sensitivity and specificity of different molecular techniques applied to the diagnosis of PJI.

**Molecular method**	**Clinical sample type tested**	**No. of samples** **tested**	**Sensitivity**	**Specificity**	**References**
Multiplex panel PCR	Sonication fluid	37	78%	na	(12)
Multiplex panel PCR	Sonication fluid	24	96%	100%	(13)
Multiplex panel PCR	Sonication fluid	144	77.1%	97.9%	(14)
Multiplex panel PCR	Periprosthetic tissue, sonication fluid	64	15.6%, 68.8%	96.8%, 100%	(15)
16S rRNA - Sanger	Sonication fluid	69	86%	93%	(16)^c^
16S rRNA – NGS (pyrosequencing)	Sonication fluid	92	90%	88%	(17)^c^
16s rRNA - Sanger	Sonication fluid	366	70%	98%	(18)^c^
16S rRNA - Sanger	Periprosthetic tissue and synovial fluid	122	68% (82%^b^)	98% (96%^b^)	(19)^c^
16S rRNA - Sanger	Biofilm^a^	157	80% (100%^b^)	94.5% (98%^b^)	(20)^c^
16S rRNA - Sanger	Periprosthetic tissue	67	75%	94%	(21)^c^
16S rRNA - Sanger	Periprosthetic tissue	264	73% (70%^b^)	95.5% (96%^b^)	(22)^c^
16S rRNA – NGS	Sonication fluid	101	(86%^b^)	(98%^b^)	(23)^c^
16S rRNA – NGS	Synovial fluid, deep tissue specimens and swabs	65	89%	73%	(24)^c^
16S rRNA – NGS	Synovial fluid	86	(87%^b^)	(82%^b^)	(25)^c^
mNGS	Sonication fluid	408	(71%^b^)^d^	(96%^b^)	(26)^c^
mNGS	Synovial fluid	168	(67%^b^)^e^	(93%^b^)	(27)^c^
mNGS	Sonication fluid	97	88%	88%	(28)
16S rRNA – Sanger, mNGS	Synovial fluid	63	82%, 96%	94%	(29)
mNGS	Synovial fluid	25	92%	92%	(30)
mNGS	Periprosthetic tissue	44	95.5%	91%	(31)
mNGS	Synovial fluid	49	96%	95%	(32)

*na, not available*.

a *Biofilm was defined as scratched samples from the surface of implants*.

b *The numbers in brackets represent calculations made by other authors in the context of a systematic review and meta-analysis (33)*.

c *The study results were included in the systematic review and meta-analysis of Li et al. (33)*.

d *In 44% of culture-negative PJI, a microorganism was identified*.

e *In 16% of culture-negative PJI, a microorganism was identified*.

The utility of NGS/mNGS in providing a clinically useful diagnosis was first demonstrated in infections of the central nervous system (38, 44). It has also been successfully applied to orthopedic infections to determine their etiology. Street et al. showed that mNGS on sonication fluid had 88% species-level sensitivity (28). The meta-analysis published by Li et al. showed that sequencing assays to diagnose PJI, including NGS-based assays, had favorable diagnostic accuracy, with a pooled sensitivity of 0.81 and a specificity of 0.94 (33). Thoendel et al., using mNGS on sonicate fluid, were able to identify new potential pathogens in 44% of culture-negative PJIs (26). Tarabichi et al. found that NGS was able to identify an organism in almost 90% of PJI cases compared with 61% for culture and to detect a potential pathogen in 80% of culture-negative PJIs. The samples included synovial fluid, deep-tissue specimens, and swabs from medullary canals. In 88% of samples, the results were concordant with culture results, and in 9 of 11 culture-negative PJIs, the authors detected three or more organisms (24). Wang et al. evaluated the efficacy, safety, accuracy, and reliability of mNGS for identifying pathogens in culture-negative PJIs. They found that antibiotic-related complications, duration of intravenous antibiotic treatment, and antibiotics costs in the mNGS-based treatment group were lower than in the empiric treatment group and yielded a favorable outcome in less time. Outcome was defined as control of the infection and absence of recurrence during follow-up (45). Compared to PCR-based assays, mNGS is a relatively young technique with plenty of potential that will inevitably improve, as demonstrated by four recent studies where its sensitivity to diagnose PJI was above 90% in one of them and 95% in three of them, respectively (29–32) (Table 1). A same-day NGS diagnostic result may significantly increase precision and efficiency while reducing the cost of PJI care. Long-read Nanopore technology, allowing real-time sequencing and analysis, seems promising to achieve same-day PJI diagnosis. Wang at al. conducted a preliminary assessment of this technology (46). The authors were able to identify the causative microorganisms of PJI within approximately 12 hours after sample collection. Furthermore, the detection of corresponding antimicrobial resistance determinants was faster compared to short-read based mNGS (46). NGS performed on cultured microorganisms isolated from a patient experiencing two episodes of PJI within a 9-month period was crucial to understand that the second episode was a new infection with the same bacterial species (47). Taken together, these studies indicate that molecular diagnostic tests in diagnosing and managing osteoarticular infection are helpful—and potentially superior to conventional culture methods—when applied in the appropriate context.

### Are We Ready for Metagenomics in Routine Diagnostics?

Multiple factors promote the implementation of NGS-based tests in routine clinical service. These include decreasing costs of NGS technology, cost savings from the replacement of other diagnostic tests, amount of information provided in a single test (prediction of virulence and drug resistance, outbreak analysis, difficult/unculturable species detection), and availability of portable rapid sequencing technology offering real-time data analysis, i.e., Nanopore Technologies (48, 49).

Because of the high sensitivity of NGS, findings must be interpreted in the clinical context, as detection of DNA is not sufficient to conclude that an identified microorganism is the cause of the infection. On the other hand, sensitivity is critically affected by the background level generated by human DNA present in the sample. Implementation of NGS-based tests in clinical settings requires standardized protocols and validation of each step, from DNA extraction and library preparation to bioinformatics analysis and validation and interpretation of sequencing results. The US Food and Drug Administration (FDA) has published general guidelines for the validation of infectious diseases with NGS-based diagnostic tests; mNGS tests have been meanwhile successfully validated by several groups in Clinical Laboratory Improvement Amendments-certified clinical laboratories (50–55). The vast number of detectable species makes it necessary to continuously monitor and independently confirm uncommon or unexpected results. Currently, for PJIs, mNGS is recommended only when patients have an inconclusive diagnosis despite the contribution of a multidisciplinary expert team, or in order to further investigate culture-negative PJIs (33, 56, 57).

## Differences in Biofilm Production

Biofilm formation is influenced by strain-specific properties. It is an interaction between environmental factors, surface structure, bacterial growth phase, and genetic determinants. Several methods have been published for the visualization or quantification of biofilm, including crystal violet staining and absorbance measurement, growth on polymethylmethacrylate beads followed by washing and sonication, scanning electron microscopy, and others. The physical and chemical surface properties in these assays may not necessarily reflect those in human infections, but they are responsible for the amount and structure of the biofilm (58). Bacteria can adhere to various metals (59) and biofilm formation may be different than on nonmetal material. However, irrespective of the method applied, several studies have demonstrated differences in biofilm production of the same species within a sample collection (60, 61). Post et al. demonstrated that, in a collection of isolates obtained from orthopedic implant-related infections, PJI isolates were more frequently strong biofilm formers than were isolates from fracture fixation-device infections (60).

The differences in biofilm production between various clones within the same species are important for personalized medicine; the transfer of these findings into clinical practice, however, is more challenging. The relation of biofilm production *in vitro* to infection manifestations in humans has not yet been established. For individual patients, it is important to know whether their infection is caused by a low or a high biofilm producer. Of note, these terms are schematic expressions without precise definition. Additional questions include whether or not there is an antimicrobial agent that is active against the causative microorganism, as well as which antibiotic concentrations are needed, and for how long, to cure the infection (62). Notably, the site of infection is in an extravascular compartment. Current strategies to tackle these questions include molecular tests for biofilm properties (section Genes Involved in Biofilm Formation and section Antimicrobial Susceptibility Testing of Biofilm Bacteria).

### Genes Involved in Biofilm Formation

The accessory gene regulator (*agr*) system in *Staphylococcus aureus* controls the expression of MSCRAMMs (microbial surface components recognizing adhesive matrix molecules) and regulates the quorum-sensing system along with the P2 and P3 promoters (63). *Agr* is required for *S. aureus* emigration from implant-associated biofilm (64). Post et al. observed statistically significant differences between orthopedic implant-related and non-implant-related infection isolates for the *sdrE, can, clfA*, and *bbp* genes (60). Thus, it is conceivable that molecular analysis may be helpful in categorizing bacterial clones into low and high biofilm producers.

Beyond the genes associated with biofilm production, bacterial toxins may interact with biofilm production, staphylococcal toxins and their impact on bone and joint infections, and factors associated with the aggregation of *S. aureus* in synovial fluid. Biofilm and exotoxin interactions have been recently reviewed elsewhere (65) and are beyond the scope of this article.

In group B streptococcus, the two-component system CovR/S regulates the expression of surface adhesion proteins (e.g., BsaB/FbsC), and CovR/S mutants show increased adherence to host cells and biofilm formation (66–68). Patras et al. (69) identified the biofilm regulatory protein A (BrpA) in group B streptococci. The carbon catabolite protein A (CcpA) is involved in the regulation of biofilm formation in oral streptococci (70). In addition, pili—long filamentous structures growing from the bacterial surface—have been associated with biofilm formation (71, 72). Genes encoding for pilus components and their development (i.e., backbone and accessory proteins, sortase enzymes) are clustered in genomic pathogenicity islands named PI-1,−2a, and−2b (73). Among these, PI-2a has been associated with the strongest biofilm-forming capacity (72).

While quantitative proteomics of strong and weak biofilm formers reveal important regulators of biofilm formers [e.g., *Enterococcus faecalis* in (74)], there is yet not a direct association of genetic elements and clinical failure. Numerous factors in the host-pathogen interaction and treatment concepts of biofilm-related infections contribute to clinical failure (75).

The constantly growing list of research findings illustrates the high potential for detecting biofilm properties via NGS in routine diagnostics. Thanks to technological advances, the entire pathogen's genome can be characterized in near real time with a sequence coverage sufficient to detect minor genetic variants, critical for directing clinical care decisions (48, 76, 77). This also allows clinical scientists to obtain a pathogen's detailed profile in terms of clone type and presence of genetic determinants associated with biofilm production.

### Antimicrobial Susceptibility Testing of Biofilm Bacteria

The minimal inhibitory concentrations (MICs) of antibiotics are routinely determined by using planktonic bacteria and do not match the concentrations that are effective in preventing, inhibiting, reducing, or eradicating biofilm bacteria (78). Different biofilm susceptibility endpoint parameters have been proposed to guide the treatment of biofilm-associated infections. These include minimal biofilm eradicating concentration (MBEC), minimal biofilm inhibitory concentration (MBIC), biofilm bactericidal concentration, and biofilm prevention concentration.

The MBEC of an antibiotic agent is the concentration of antibiotic needed to kill all viable bacteria within an established biofilm, including persister cells. MBEC determination is not offered in clinical microbiology diagnostic laboratories, it is not standardized or validated to be performed routinely, and antibiotic exposure time is not reported. Methods for MBEC determination have technical difficulties, leading to considerable variability in results (79, 80). Furthermore, biofilm age is an important factor; thus, MBEC depends on the point of readout (e.g., 24 vs. 120 hours) (81). Host factors such as plasma and heme increase tolerance to antibiotics. The same aged biofilm in normal media vs. media with human plasma has been shown to have up to a 100-fold difference in tolerance (82). This again illustrates the importance of considering differences between *in vivo* and *in vitro* when interpreting MBEC results. For Gram-positive organisms, MBECs of beta-lactams and glycopeptides are typically several 100 to 1000 times higher than the corresponding MIC of planktonic bacteria (62). The MBEC:MIC ratio for aminoglycosides and rifampin is typically lower than are those for beta-lactams. However, *Staphylococcus* spp., *Streptococcus* spp., and *Enterococcus* spp. may display high-level gentamicin resistance (62, 83).

For MBECs to provide a clinically useful result, a standardized assay is needed, in particular when considering antibiotic therapy as part of personalized medicine (84). Consequently, interest in antimicrobial susceptibility testing in biofilms is ongoing, and several methods have been implemented in the last few years (78).

Many published results derive from the MBEC device (formerly called the Calgary device, Innovotech Inc., Edmonton, Alberta, Canada) (85). The method is challenging, as it requires a specific protocol for every species and a antimicrobial agent [supplementary material in (85)]. Recently, a promising steam-based method has been developed by Tasse et al. (86), showing similar results to those of the MBEC device. This easy-to-handle and easy-to-implement method points toward interinstitutional comparability of MBEC results. However, there is a lack of data and direct link between MBEC results and clinical outcome, and hence, there is no scientific reasoning to favor one of the methods.

The challenge reoccurs when transferring MBEC results to antibiotic dosing in patients and treatment concept. It may seem logical that high MBEC results are associated with high biofilm production, failure to achieve the required antibiotic concentration at the site of infection, and hence, clinical failure. Conversely, low MBEC results may be associated with low biofilm production, success in achieving the required antibiotic concentration at the site of infection, and considerable chance of clinical cure. Unfortunately, there is no such simple linearity because many technical, material, and environmental factors influence the microbiological result, as outlined earlier. Nonetheless, a similar concept was recently applied on a case basis in a context other than orthopedic infections, namely, in a patient with a cardiac device-related infection and conduit valve prosthesis endocarditis caused by nutritionally variant streptococci (87). Biofilm production was investigated on three different materials (bone cement, glass, plastic), and several different culture media. Biofilm eradication concentrations were examined with two different methods. All tests uniformly demonstrated that the causative microorganism did not produce biofilm. The MBEC results were similar to MIC results. Conservative treatment without device removal proved to be successful. Although the example is only a single case, it raises (at least) three thoughts. Firstly, these concepts require fruitful collaboration of many disciplines in a joint team effort. Secondly, the number of required investigations for a single case is still too high for immediate transfer to routine clinical practice. And thirdly, depending on the complexity and specific circumstances of a clinical case requiring treatment decision making, the hypothesized linearity of low MBEC results and low biofilm production may be more tempting to believe than the presumed association of high MBEC results and high biofilm production.

## Perspective: Precision Medicine to Aid Decision Making in Orthopedic Biofilm Infections

The concept of considering clone-level (rather than only species-level) bacterial properties in the medical decision-making process represents a promising perspective. The more invasive the surgical procedure for removing a device, the higher the complication rate. If we are able to identify microorganisms with poor biofilm production by using reliable methods prior to the planned surgical intervention, patients could potentially benefit from conservative treatment. Conversely, unsuccessful attempts at implant retention could be avoided in the presence of microorganisms with strong biofilm production. Lack of clonal analysis beyond species identification for predicting outcome in established treatment concepts reflects a knowledge gap, and findings supporting this approach will affect further research beyond the field of septic surgery. Implementation of such a concept will have cost-saving effects, considering the duration of hospitalization expenditures associated with avoidable surgical infections and the complications associated with these interventions.

## Data Availability Statement

The original contributions presented in the study are included in the article/supplementary material, further inquiries can be directed to the corresponding author/s.

## Author Contributions

RB drafted and co-wrote the manuscript and performed the literature review. PS revised the manuscript, supervised the work, and co-wrote the manuscript. Both authors contributed to the article and approved the submitted version.

## Conflict of Interest

The authors declare that the research was conducted in the absence of any commercial or financial relationships that could be construed as a potential conflict of interest.
